# A novel imatinib-upregulated long noncoding RNA plays a critical role in inhibition of tumor growth induced by *Abl* oncogenes

**DOI:** 10.1186/s12943-021-01478-5

**Published:** 2022-01-03

**Authors:** Yun Ma, Guijie Guo, Tingting Li, Faxin Wen, Jianling Yang, Biao Chen, Xuefei Wang, Ji-Long Chen

**Affiliations:** 1 CAS Key Laboratory of Pathogenic Microbiology and Immunology, Institute of Microbiology, Chinese Academy of Sciences (CAS), Beijing, 100101 China; 2 University of Chinese Academy of Sciences, Beijing, 100049 China; 3 Key Laboratory of Fujian-Taiwan Animal Pathogen Biology, College of Animal Sciences, Fujian Agriculture and Forestry University, Fuzhou, 350002 China; 4 Institute of Medical Innovation and Research, Peking University Third Hospital, Beijing, 100191 China

**Keywords:** lncRNA-IUR1, lncRNA, Bcr-Abl, Cellular transformation, Leukemia

## Abstract

**Background:**

Dysregulation of long noncoding RNAs (lncRNAs) has been linked to various human cancers. *Bcr-Abl* oncogene that results from a reciprocal translocation between human chromosome 9 and 22, is associated with several hematological malignancies. However, the role of lncRNAs in Bcr-Abl-induced leukemia remains largely unexplored.

**Methods:**

LncRNA cDNA microarray was employed to identify key lncRNAs involved in Bcr-Abl-mediated cellular transformation. Abl-transformed cell survival and xenografted tumor growth in mice were evaluated to dissect the role of imatinib-upregulated lncRNA 1 (IUR1) in Abl-induced tumorigenesis. Primary bone marrow transformation and in vivo leukemia transplant using lncRNA-IUR1 knockout (KO) mice were further conducted to address the functional relevance of lncRNA-IUR1 in Abl-mediated leukemia. Transcriptome RNA-seq and Western blotting were performed to determine the mechanisms by which lncRNA-IUR1 regulates Bcr-Abl-induced tumorigenesis.

**Results:**

We identified lncRNA-IUR1 as a critical negative regulator of Bcr-Abl-induced tumorigenesis. LncRNA-IUR1 expressed in a very low level in Bcr-Abl-positive cells from chronic myeloid leukemia patients. Interestingly, it was significantly induced in Abl-positive leukemic cells treated by imatinib. Depletion of lncRNA-IUR1 promoted survival of Abl-transformed human leukemic cells in experiments in vitro and xenografted tumor growth in mice, whereas ectopic expression of lncRNA-IUR1 sensitized the cells to apoptosis and suppressed tumor growth. In concert, silencing murine lncRNA-IUR1 in Abl-transformed cells accelerated cell survival and the development of leukemia in mice. Furthermore, lncRNA-IUR1 deficient mice were generated, and we observed that knockout of murine lncRNA-IUR1 facilitated Bcr-Abl-mediated primary bone marrow transformation. Moreover, animal leukemia model revealed that lncRNA-IUR1 deficiency promoted Abl-transformed cell survival and development of leukemia in mice. Mechanistically, we demonstrated that lncRNA-IUR1 suppressed Bcr-Abl-induced tumorigenesis through negatively regulating STAT5-mediated GATA3 expression.

**Conclusions:**

These findings unveil an inhibitory role of lncRNA-IUR1 in Abl-mediated cellular transformation, and provide new insights into molecular mechanisms underlying Abl-induced leukemogenesis.

**Supplementary Information:**

The online version contains supplementary material available at 10.1186/s12943-021-01478-5.

## Background

Chronic myeloid leukemia (CML) is a hematological malignancy mainly caused by the *Bcr-Abl* oncogene that results from the reciprocal translocation between human chromosome 9 and 22, and occurs in more than 90% of CML cases [1]. *Bcr-Abl* oncogene is also involved in tumorigenesis of other leukemia such as acute lymphoid leukemia (ALL) [2]. *v-Abl* is the oncogene of Abelson murine leukemia virus, which contributes to the malignant transformation of mouse pre-B cells and lymphoid tumorigenesis in mice [3]. Owing to their constitutive tyrosine kinase activity, Abl oncoproteins (Bcr-Abl, v-Abl) activate a variety of signaling pathways associated with cell survival and proliferation, such as PI3K/AKT (phosphatidylinositol 3-kinase/protein kinase B) and JAK/STAT (Janus kinase/signal transducer and activator of transcription), resulting in uncontrolled cell survival and proliferation, and the development of leukemia [4–6]. Although progress has been made in the understanding of signal transduction in Abl-mediated transformation, the molecular mechanisms underlying Abl-induced tumorigenesis are still not fully understood. On the other hand, it is well known that imatinib treatment can greatly suppress Bcr-Abl-mediated development of leukemia, but intracellular molecules induced by imatinib and their roles in the tumorigenesis remain largely to be determined.

The majority of human transcripts lack protein-coding capacity, which are defined as noncoding RNAs (ncRNAs) [7]. Long noncoding RNAs (lncRNAs) are the ncRNAs longer than 200 nt without protein-coding capacity or with micropeptide-coding capacity [8–10]. Accumulating studies have revealed that lncRNAs play important roles in various vital biological processes [11–14]. Notably, increasing number of lncRNAs have been linked to human diseases, including cancers [15–19]. For instance, upregulation of lncRNA SLCC1 promotes colorectal carcinogenesis through activating the glycolysis pathway [20]. Depletion of lncRNA XIST represses the progression of ovarian cancer cell by upregulating the expression of microRNA-149-3p [21]. Upregulation of lncRNA metastasis-associated lung adenocarcinoma transcript 1 (MALAT1) was observed in persistence triple-negative breast cancer (TNBC) patients compared with that in extinction TNBC patients in response to neoadjuvant chemotherapy (NAC), suggesting a role of MALAT1 in conferring TNBC resistance to NAC therapy [22]. Additionally, lncRNAs may represent an attractive target for the diagnosis and treatment of cancers. LncRNA PCA3 is released in the urine of Prostate cancer (PCa) patients and has been shown to be a less invasive, more specific and sensitive marker for PCa than the currently used serum prostate-specific antigen [23]. LncRNA RP11-445H22.4 is overexpressed in breast cancer and could be detected in serum samples with high sensitivity and specificity, indicating that lncRNA RP11-445H22.4 may be a promising diagnostic marker for breast cancer [24]. Intriguingly, numerous lncRNAs, such as urothelial carcinoma associated 1 (UCA1), antisense noncoding RNA in the INK4 locus (ANRIL), maternally expressed 3 (MEG3), LAMP5 antisense 1 (LAMP5-AS1), and nuclear paraspeckle assembly transcript 1 (NEAT1) have been reported to be associated with leukemogenesis [25–31]. However, the biological significance and underlying mechanisms of various lncRNAs in the development of leukemia remain poorly characterized.

Our group has previously identified several functional lncRNAs involved in Bcr-Abl-induced oncogenic transformation. For example, we identified lncRNA beta globin locus 3 (BGL3) as a critical tumor suppressor in Bcr-Abl-mediated tumorigenesis. LncRNA-BGL3 can act as a competitive endogenous RNA (ceRNA) to positively regulate the expression of phosphatase and tensin homolog (PTEN), thereby promoting Abl-transformed leukemic cell apoptosis and suppressing xenografted tumor growth in vivo [32]. In addition, we found that H19 was highly expressed in Bcr-Abl-transformed cell lines and primary cells derived from patients, and promoted efficient tumorigenesis induced by Bcr-Abl [33]. Recently, we identified a conserved imatinib-upregulated lncRNA (IUR) as a negative regulator of Bcr-Abl-induced tumorigenesis. Low expression of lncRNA-IUR was detected in the peripheral blood lymphocytes derived from Bcr-Abl-positive leukemia patients. Loss of murine lncRNA-IUR promoted Bcr-Abl-mediated primary bone marrow transformation, Abl-transformed leukemic cell survival and Abl-mediated mouse leukemia [34]. Despite their importance, a large fraction of lncRNAs implicated in cellular transformation by Abl oncogenes remains unexplored and deserves further investigation.

In the current study, we identified a novel imatinib-upregulated lncRNA, designated lncRNA-IUR1 that critically regulated Abl-mediated cellular transformation. The expression of lncRNA-IUR1 was highly induced by imatinib treatment in Abl-transformed leukemic cells. Loss of lncRNA-IUR1 promoted leukemic cell survival and tumor growth in mice. Knockout of lncRNA-IUR1 in mice facilitated Abl-mediated transformation of primary bone marrow cells and the progression of Abl-mediated leukemia in mice. Furthermore, we revealed that lncRNA-IUR1 suppressed Abl-induced tumorigenesis through regulation of STAT5-mediated GATA3 expression. These findings highlight the functional involvement and physiological significance of lncRNAs in Abl-mediated oncogenic transformation, and provide new insights into complicated mechanisms underlying hematopoietic malignancies.

## Materials and methods

### Ethics statement

The mouse experimental design and protocol used in this study were approved by the Regulation of the Institute of Microbiology, Chinese Academy of Sciences of Research Ethics Committee (Permit Number: SQIMCAS2018043). All mouse experiments were performed in accordance with the Regulations for the Administration of Affairs Concerning Experimental Animals approved by the State Council of People’s Republic of China. All participants signed informed consent prior to using the peripheral blood cells for scientific research.

### Microarray and RNA-seq analysis

The lncRNA cDNA microarray was from Agilent (Santa Clara, CA, USA). Total RNAs from three independent groups of K562 cells treated with imatinib or control cells were prepared using Trizol reagent (Invitrogen, Carlsbad, CA, USA). Sample labeling, hybridization and data analysis were performed as previously described [34]. The microarray data have been deposited in the NCBI Gene Expression Omnibus (Accession number GSE119770).

Total RNAs isolated from three independent groups of K562 cells expressing shRNA targeting lncRNA-IUR1 or control shRNA, were used for RNA-seq. RNA libraries were prepared for sequencing using standard Illumina protocols. Illumina Casava software was employed for basecalling during the data procession. Sequenced reads were trimmed for adaptor sequence, masked for low-complexity or low-quality sequence, and then mapped to hg38 whole genome. Reads Per Kilo bases per Million reads (RPKM) were calculated and analyzed using samtools v0.1.19. RNA-seq data have been deposited on GEO public database (Accession number GSE181535).

### Cell lines and cell culture

K562 and HEK293T cell lines were purchased from ATCC (American Type Culture Collection) (Manassas, VA, USA). The v-Abl-transformed mouse cell lines NS2 and W44 were generated as described previously [4, 5]. Cells were cultured with DMEM or RPMI 1640 supplemented with 10% fetal bovine serum (FBS).

### DNA transfection, viral packaging and lentiviral infection

DNA transfection was performed using VigoFect (Vigorous Biotechnology). Lentiviruses expressing shRNAs were packaged in HEK293T cells in which shRNA constructs, and lentiviral packaging plasmids (PLP1, PLP2 and PLP-VSVG) were co-transfected. Lentiviral vector pNL/EGFP/CMV/WPREΔU3 (41790, Addgene) was utilized for overexpression experiments. LncRNA-IUR1 or lncRNA-mIUR1 cDNA was cloned into the lentiviral vector using Nhe1 and Xho1 sites. The lncRNA-IUR1 or lncRNA-mIUR1 cDNA construct along with lentiviral packaging vectors were transfected into HEK293T cells. For the generation of stable lncRNA-IUR1 knockdown or overexpressing cells, the virus-containing supernatant was collected 48 h after transfection and passed through a 0.45 μm filter to eliminate cells. Cells in 6-well tissue culture plates were infected with the virus in medium containing 8 μg ml^− 1^ of polybrene and a spin infection was performed by centrifugation at 2200 rpm for 2 h.

### RNA interference

The following shRNAs were used in this study:

- human lncRNA-IUR1 shRNA: 5′-GCTCTTCAGTTCTGACCTTCC-3′,
- mouse lncRNA-IUR1 shRNA: 5′-GTGCATACAGTAGGCCTAGCA-3′,
- mouse GATA3 shRNA: 5′-GCAATGCCTGCGGACTCTACC-3′.

### Reverse transcription PCR (RT-PCR) and quantitative real-time PCR

RNA was isolated with TRIzol RNA Isolation Reagents (Thermo Fisher). Reverse transcription was performed with PrimeScript™ RT Reagent Kit (Takara), and quantitative real-time PCR was performed with Power SYBR Green Master Mix (Thermo Fisher). For quantification, the 2^−ΔΔCt^ method was used to calculate the relative RNA levels against GAPDH or β-Actin. The peripheral blood cells were treated with red blood cell lysis buffer to remove the red blood cells, and then subjected to RNA isolation followed by RT-PCR.

### 5′ and 3′ RACE

5′ and 3′ RACE experiments were performed using the SMARTer RACE cDNA amplification Kit (Clontech, Mountain View, CA, USA) according to the manufacturer’s instruction.

### Cell apoptosis assay

Cell apoptosis assay was performed as previously described [32]. Briefly, cells were treated with imatinib for indicated time, harvested and stained with propidium iodide (PI)/Annexin V, and then analyzed by fluorescence activated cell sorter (FACS) (BD Biosciences, San Jose, CA, USA). TUNEL assay was performed using the TUNEL kit (Beyotime) according to the manufacturer’s instruction.

### Cell cytotoxicity and colony formation assays

Cell cytotoxicity assay was performed using the CCK-8 kit (Beyotime) according to the manufacturer’s instruction. For colony formation assay, K562 cells were washed with PBS and plated in methylcellulose (STEMCELL). Colonies were counted 7-10 days later.

### Immunofluorescence

For immunostaining experiment, fresh tissues were embedded in optimal cutting temperature compound, sectioned onto slides and fixed with 4% paraformaldehyde for 15 min as previously described [35]. Tissues were then permeabilized with 0.5% Triton X-100, incubated with primary antibody and subsequently incubated with the corresponding secondary antibody. Cellular nuclei were stained with DAPI. The coverslips were mounted onto glass slides with anti-fade solution and visualized using the fluorescence microscope.

### Tumor xenograft

Nude mice injection was performed as previously described [36]. In brief, K562 or NS2 cells were injected subcutaneously into the flanks of 4-5 weeks old female athymic nude Nu/Nu mice. Tumor growth was monitored, and tumor volume was measured and calculated using the formula (length × width × height)/2 at the indicated time after inoculation. Bioluminescent imaging was performed to detect tumors from GFP-expressing cells.

### In vivo leukemia transplant

Leukemia transplantation was performed as previously described [34]. GFP-positive NS2 cells (1 × 10^7^) were injected into sub-lethally (5.5 Gy, X-ray) irradiated recipients (C57BL/6 N mice) through tail vein.

### LncRNA-IUR1 knockout mice

Murine lncRNA-IUR1 knockout mice were generated by CRISPR/Cas9-based genome editing system as previously described [37]. Briefly, two sgRNAs targeting 5′ end (gRNA 1: AACCTTTGAGTCTCTCAGTC) and 3′ end (gRNA 2: TATCATTTGTAGAACTAGGC) of murine lncRNA-IUR1 were respectively constructed and transcribed in vitro. The Cas9 mRNA and sgRNAs were co-injected into zygotes. The zygotes were then transferred into the oviduct of pseudo-pregnant ICR females at 0.5 dpc to farrow litters. The litters were genotyped by PCR. The primers used for lncRNA-mIUR1 genotyping were: KO-mIUR1-F: 5′- GATGATCCTAACCTTCTCTGAGCTG − 3′, KO-mIUR1-R: 5′- TGACCTGCTACCTGCTATGTAGTC − 3′, and WT-F: 5′- TTGCATCCTGCTCTACGTTCTTAGC − 3′.

### Primary bone marrow transformation assay

Primary bone marrow transformation was carried out as previously described [32]. Transformation efficiency was measured by counting the number of Bcr-Abl-transformed cell clones.

### Data analysis

Data are presented as mean ± SEM of at least three independent experiments. Statistical analyses were performed in Microsoft Excel 2010 with the Student’s two-tailed *t* test, and *p* value < 0.05 was considered to be significant. Statistical significance is represented in figures by: *P < 0.05; **P < 0.01; ***P < 0.001.

## Results

### LncRNA-IUR1 is a novel lncRNA whose expression is low in Bcr-Abl-positive leukemic cells but highly induced by imatinib

**Fig. 1:**
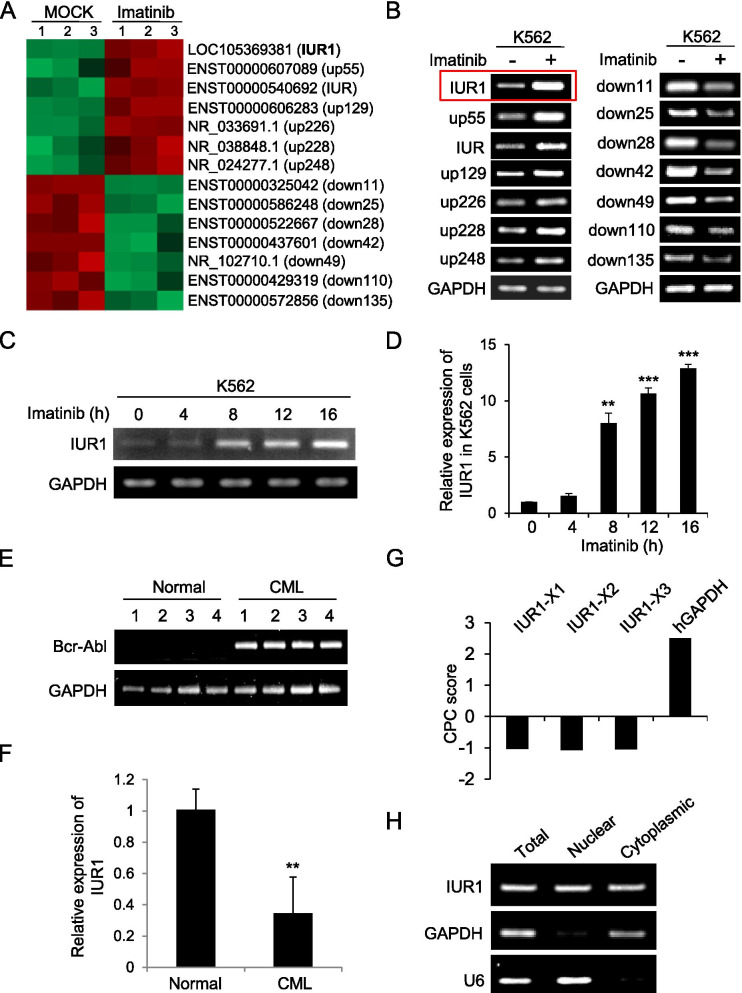
**LncRNA-IUR1 is identified as a novel lncRNA that is induced by imatinib treatment in Bcr-Abl-positive leukemic cells. A, B** Hierarchical clustering analysis of lncRNAs differentially expressed in K562 cells treated with or without imatinib. The values from three independent experiments were displayed (**A**). RT-PCR analysis of expression of selected lncRNAs in K562 cells treated with imatinib (**B**). **C**, **D** RT-PCR (**C**) and quantitative real-time PCR (**D**) were performed to detect the expression of lncRNA-IUR1 in K562 cells treated with imatinib. Data (**D**) are presented as mean ± SEM. *n* = 3, **p < 0.01, ***p < 0.001. **E**, **F** RT-PCR analysis of Bcr-Abl expression in the primary peripheral blood lymphocytes from Bcr-Abl-positive CML patients and normal subjects (**E**). Quantitative real-time PCR was performed to examine the expression of lncRNA-IUR1 in primary leukemic cells from Bcr-Abl-positive CML patients and normal control (**F**)**.** *n* = 4, means ± SEM, **p < 0.01. **G** The CPC scores for human lncRNA-IUR1 transcripts and the control gene GAPDH (http://cpc2.cbi.pku.edu.cn/). The CPC score of each lncRNA-IUR1 transcript was minus, indicating “noncoding”. **H** RT-PCR was performed to examine cytoplasmic or nuclear lncRNA-IUR1 RNA levels in K562 cells. GAPDH was served as the cytoplasmic control, and U6 was the nuclear control

To identify key long noncoding RNAs (lncRNAs) involved in Bcr-Abl-mediated tumorigenesis, an lncRNA microarray was employed to analyze the expression of lncRNAs in Bcr-Abl–positive K562 cells treated with or without the Abl kinase inhibitor imatinib. Numerous lncRNAs were found to display differential expression in response to imatinib treatment (Fig. 1A, B). Notably, the expression of a novel lncRNA, designated as imatinib-upregulated lncRNA 1 (IUR1), was highly induced by imatinib treatment (Fig. 1A-D). Intriguingly, lncRNA-IUR1 was expressed in a very low level in Bcr-Abl-positive cells from chronic myeloid leukemia patients as compared with that in normal subjects (Fig. 1E, F), which prompted us to explore the role of lncRNA-IUR1 in Bcr-Abl-induced tumorigenesis.

LncRNA-IUR1 is located between the *purinergic receptor P2Y2 (P2ry2)* gene and the *FCH and double SH3 domains 2 (Fchsd2)* gene on human chromosome 11 (Supplementary Fig. S1A-B). Next, we analyzed the protein-coding potential of lncRNA-IUR1 through software prediction and experiments. As shown in Fig. 1G, the coding potential calculator (CPC) analysis showed that the CPC score of each lncRNA-IUR1 transcript was minus, indicating “noncoding”. Using in vitro translation experiments, we failed to detect any specific protein band for lncRNA-IUR1 while a specific protein band (about 72 kD) was observed for the control gene gag (Supplementary Fig. S1C), supporting that lncRNA-IUR1 had no protein-coding capability. In addition, we examined the localization of lncRNA-IUR1 in Bcr-Abl-positive leukemic cells by subcellular fractionation analysis, and found that lncRNA-IUR1 was localized in both cytoplasm and nucleus (Fig. 1H).

### Altering lncRNA-IUR1 expression affects Bcr-Abl-transformed cell survival in vitro and tumor growth in vivo

**Fig. 2:**
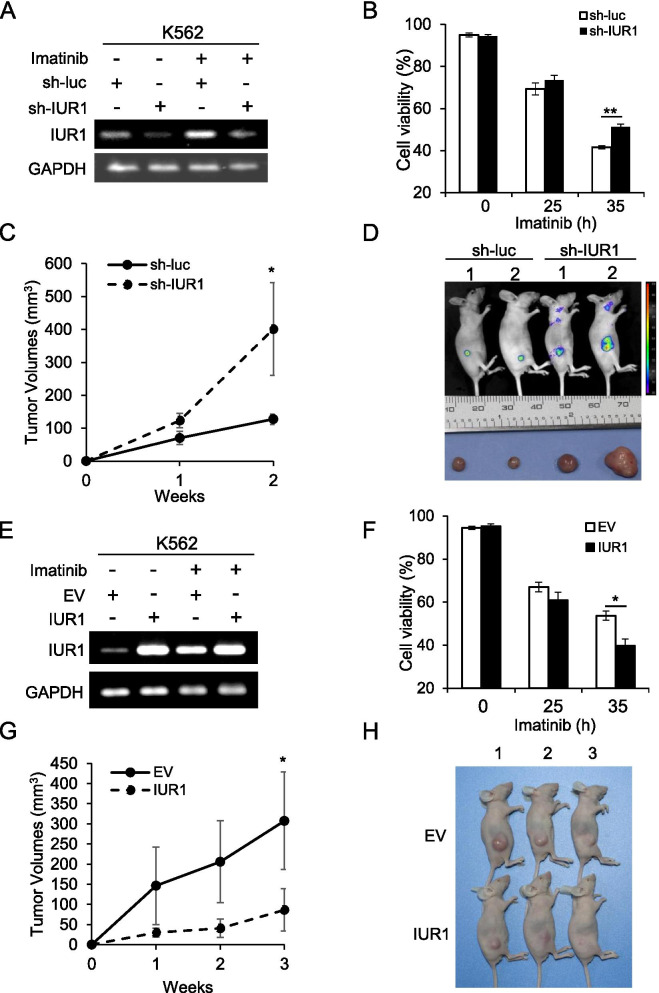
**Altering lncRNA-IUR1 expression regulates Bcr-Abl-transformed cell survival in vitro and tumor growth in vivo*.*** **A** RT-PCR was performed to examine lncRNA-IUR1 RNA levels in K562 cells stably expressing control (sh-luc) or lncRNA-IUR1 shRNA (sh-IUR1) respectively with or without imatinib treatment. **B** Cell survival analysis of control and lncRNA-IUR1 knockdown K562 cells upon imatinib treatment (5 μM). Data are presented as mean ± SEM. *n* = 3, **p < 0.01. **C**, **D** Nude mice were subcutaneously injected with control or lncRNA-IUR1 knockdown K562 cells (**C**). Tumor growth was monitored by bioluminescent imaging, and tumors were excised from the nude mice. Shown were representative images from at least three independent experiments (**D**). Data are presented as mean ± SEM. *n* = 3, *p < 0.05. **E** RT-PCR was performed to examine lncRNA-IUR1 expression in K562 cells stably expressing empty vector (EV) or lncRNA-IUR1 (IUR1) respectively with or without imatinib treatment. **F** Cell viability of control and lncRNA-IUR1 overexpressing K562 cells, was analyzed in response to imatinib treatment (5 μM). Data are presented as mean ± SEM. n = 3, *p < 0.05. **G**, **H** Nude mice were subcutaneously injected with control or lncRNA-IUR1 overexpressing K562 cells. Tumor growth was monitored as described above (**G**). Shown were representative images from at least three independent experiments (**H**). Data are presented as mean ± SEM. n = 3, *p < 0.05

To dissect the role of lncRNA-IUR1 in Bcr-Abl mediated cellular transformation, we first evaluated the effect of altered lncRNA-IUR1 expression on Bcr-Abl-transformed cell survival. LncRNA-IUR1 knockdown K562 cells stably expressing lncRNA-IUR1 shRNA and control cells were generated, treated with imatinib, and subjected to cell survival analysis (Fig. 2A). We observed that disruption of lncRNA-IUR1 expression in K562 cells resulted in a significant increase in viable cells compared with the control cells after treatment with imatinib (Fig. 2B, Supplementary Fig. S2A-D), while no significant difference in cell cycle progression was found between control and lncRNA-IUR1 knockdown cells (Supplementary Fig. S2E). Consistently, lower levels of cleaved caspase-3, caspase-9 and PARP were observed in lncRNA-IUR1 depleted K562 cells than those in control cells after imatinib treatment (Supplementary Fig. S2F-G). The data suggest that knockdown of lncRNA-IUR1 promotes Bcr-Abl-positive cell survival in response to imatinib treatment. Then, we further examined the effect of lncRNA-IUR1 deficiency on Bcr-Abl-induced tumorigenesis in vivo. Control or lncRNA-IUR1 knockdown K562 cells were injected into nude mice subcutaneously, and tumor growth was examined. It was shown that tumors formed by lncRNA-IUR1 knockdown cells grew much faster than that formed by control cells (Fig. 2C, D). Analysis of Ki-67 expression in the tumors revealed that the level of Ki-67 was elevated in tumors formed by lncRNA-IUR1 depleted K562 cells than those formed by control cells, while a lower degree of apoptosis was observed in tumors formed by lncRNA-IUR1 knockdown K562 cells than those by the control cells (Supplementary Fig. S2H-K). These results demonstrate that silencing of lncRNA-IUR1 promotes Bcr-Abl-transformed leukemia cell survival in vitro and tumor growth in vivo.

On the other hand, we examined the effect of lncRNA-IUR1 overexpression on Bcr-Abl-induced tumorigenesis. LncRNA-IUR1 overexpressing K562 cells and control cells were generated, and analyzed for cell survival in response to imatinib treatment (Fig. 2E). In contrast to the promotion of Abl-positive cell survival evoked by lncRNA-IUR1 deficiency, enforced expression of lncRNA-IUR1 sensitized K562 cells to imatinib-induced apoptosis while had no significant effect on cell cycle progression (Fig. 2F, Supplementary Fig. S2L). Accordingly, overexpression of lncRNA-IUR1 remarkably impeded the growth of K562 cell xenografts in nude mice (Fig. 2G, H, Supplementary Fig. S2M). Collectively, these results demonstrate that lncRNA-IUR1 plays an inhibitory role in Bcr-Abl-induced tumorigenesis.

### Identification of murine lncRNA-IUR1

**Fig. 3:**
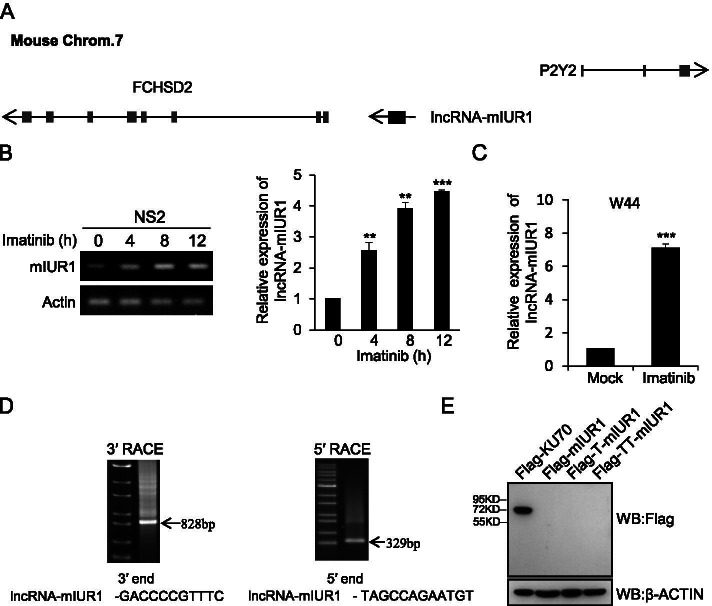
**Identification of murine lncRNA-IUR1. A** A schematic representation of the genomic location of murine lncRNA-IUR1 and its adjacent genes *P2ry2* and *Fchsd2*. The orientation of arrows indicated the transcription direction. **B** RT-PCR (left) and quantitative real-time PCR (right) were performed to detect the expression of murine lncRNA-IUR1 in NS2 cells treated with imatinib. Data of quantitative real-time PCR (right) are presented as mean ± SEM. n = 3, **p < 0.01, ***p < 0.001. **C** Quantitative real-time PCR analysis of the RNA levels of murine lncRNA-IUR1 in W44 cells treated with imatinib. Data are presented as mean ± SEM. n = 3, ***p < 0.001. **D** Agarose gel analysis of 5′ RACE and 3′ RACE PCR products of murine lncRNA-IUR1. 5′ and 3′ end sequences were show in below. **E** Murine lncRNA-IUR1 was cloned into pNL vector with an N-terminal Flag tag in three reading frames. The constructs were transfected into HEK293T cells for 48 h. Cell lysates were harvested and subjected to Western blotting with Flag antibody. Flag-KU70 was served as a positive control

Since human lncRNA-IUR1 critically regulates Bcr-Abl-mediated cellular transformation, this prompted us to identify murine homologous lncRNA-IUR1, which may help to further address the functional relevance of lncRNA-IUR1 in Abl-induced tumorigenesis under a more sophisticated and physiological circumstance. Sequence alignment analysis between human lncRNA-IUR1 transcript and the mouse genome, revealed a 442 bp mouse genome sequence with up to 72% homology to human lncRNA-IUR1, which we call lncRNA-mIUR1 (murine lncRNA-IUR1) (Supplementary Fig. S3A). Of particular interest, lncRNA-mIUR1 is located between the *P2ry2* gene and the *Fchsd2* gene on mouse chromosome 7 (Fig. 3A), which displays highly concordant genomic location with human lncRNA-IUR1 (Supplementary Fig. S1A), indicating that lncRNA-mIUR1 is probably the murine homologue of lncRNA-IUR1.

Then, we performed reverse transcriptase PCR and quantitative real-time PCR to examine the expression of lncRNA-mIUR1 transcript in response to imatinib treatment. Indeed, the expression of lncRNA-mIUR1 transcript significantly increased after imatinib treatment in v-Abl-transformed mouse cells (Fig. 3B, C). This is consistent with the upregulation of human lncRNA-IUR1 induced by imatinib in human Bcr-Abl-positive cells. These experiments support that lncRNA-mIUR1 is the murine homologous lncRNA-IUR1 and Abl-mediated regulation of lncRNA-IUR1 is evolutionally conserved across species. 5′ RACE and 3′ RACE experiments were performed to determine the full length of lncRNA-mIUR1. The full length of lncRNA-mIUR1 is 2257 nt and the sequence has been submitted to GenBank (MZ643464) (Fig. 3D).

Next, we analyzed the protein-coding potential of murine lncRNA-IUR1 transcript through software prediction and experimental study. As shown in Supplementary Fig. S3B-C, the coding potential calculator (CPC) and open reading frame (ORF) Finder analysis demonstrated that lncRNA-mIUR1 had no protein-coding capacity. Besides, using in vitro translation experiments, we failed to detect any specific protein band for lncRNA-mIUR1 while a specific protein band (about 70 kD) was observed for the control gene KU70 (Fig. 3E), supporting that lncRNA-mIUR1 is a non-coding RNA.

### Murine lncRNA-IUR1 regulates v-Abl-transformed cell survival and tumor growth

**Fig. 4:**
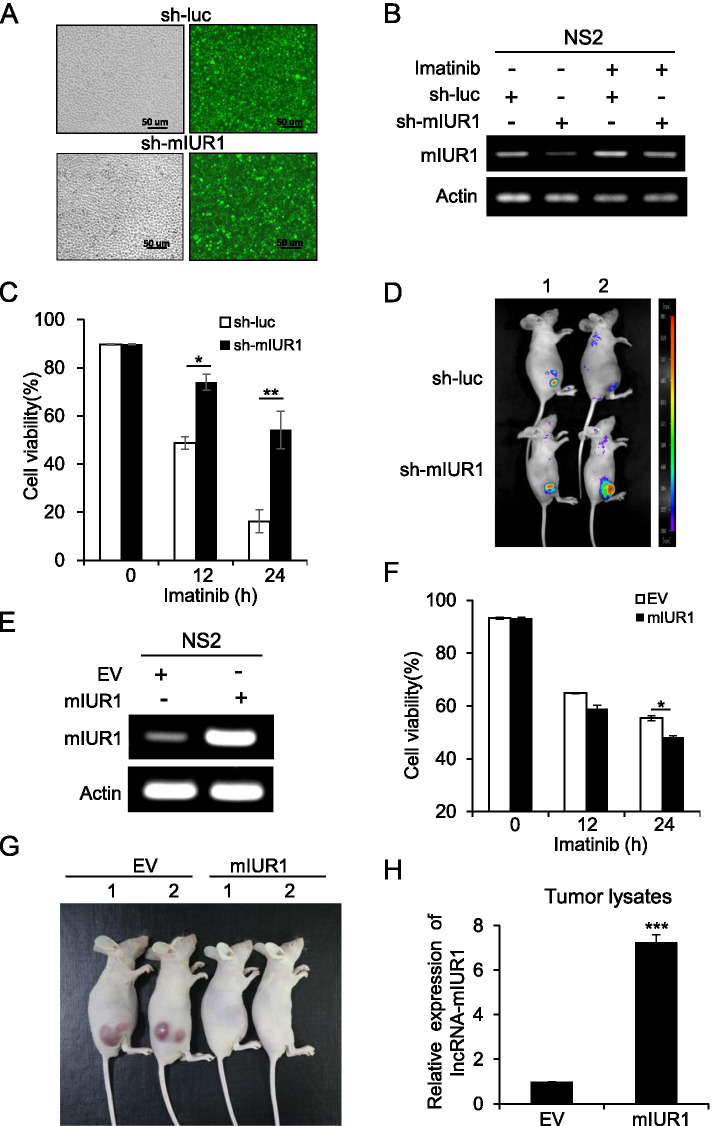
**Altering murine lncRNA-IUR1 expression regulates v-Abl-transformed cell survival in vitro and tumor growth in vivo*.*** **A**, **B** Control and lncRNA-IUR1 knockdown NS2 cells were generated. NS2 cells were infected with GFP-positive lentiviruses harboring control (sh-luc) or murine lncRNA-IUR1 shRNA (sh-mIUR1) respectively. GFP-positive NS2 cells were sorted by FACS. Shown were representative micrographs of the generated cells (**A**). RT-PCR was performed to examine the expression of lncRNA-IUR1 in NS2 cells stably expressing sh-luc or sh-mIUR1 with or without imatinib treatment (**B**). **C** Cell viability of control and lncRNA-IUR1 knockdown NS2 cells was analyzed by flow cytometry upon treatment with imatinib (2.5 μM). Data are presented as mean ± SEM. *n* = 3, *p < 0.05, **p < 0.01. **D** Nude mice were subcutaneously injected with control or murine lncRNA-IUR1 knockdown NS2 cells. Tumor growth was measured by bioluminescent imaging. Shown were representative images from at least three independent experiments. **E** RT-PCR was performed to examine the expression of lncRNA-IUR1 in NS2 cells stably expressing empty vector (EV) or murine lncRNA-IUR1 (mIUR1). **F** Cell survival analysis of control and lncRNA-IUR1 overexpressing NS2 cells in response to imatinib treatment (2.5 μM). Data are presented as mean ± SEM. *n* = 3, *p < 0.05. **G**, **H** Nude mice were subcutaneously injected with control or lncRNA-IUR1 overexpressing NS2 cells. Tumor growth was monitored. Shown were representative images from at least three independent experiments (**G**). The expression of lncRNA-IUR1 in tumors fromed by control or lncRNA-IUR1 overexpressing NS2 cells, was analyzed by quantitative real-time PCR (**H**). Data (**H**) are presented as mean ± SEM. *n* = 3, ***p < 0.001

We next probed whether murine lncRNA-IUR1 also plays an important role in Abl-mediated cellular transformation. We generated control and lncRNA-mIUR1 knockdown v-Abl-transformed NS2 cells, and examined the effect of lncRNA-mIUR1 deficiency on Abl-induced tumorigenesis. We observed that depletion of murine lncRNA-IUR1 in NS2 cells promoted cell survival in vitro and xenografted tumor growth in vivo (Fig. 4A-D). By contrast, overexpression of lncRNA-mIUR1 in NS2 cells dampened cell survival and tumor growth in mice (Fig. 4E-H). Overall, in line with the inhibitory role of human lncRNA-IUR1 in Bcr-Abl-mediated cellular transformation, these results demonstrate that murine homologous lncRNA-IUR1 also suppresses tumorigenesis induced by v-Abl oncogene. Together, these data suggest that lncRNA-IUR1 might be an evolutionarily conserved lncRNA that has critical functions in tumorigenesis induced by Bcr-Abl and v-Abl oncogenes.

### Depletion of murine lncRNA-IUR1 in Abl-transformed cells promotes the development of leukemia in mice

**Fig. 5:**
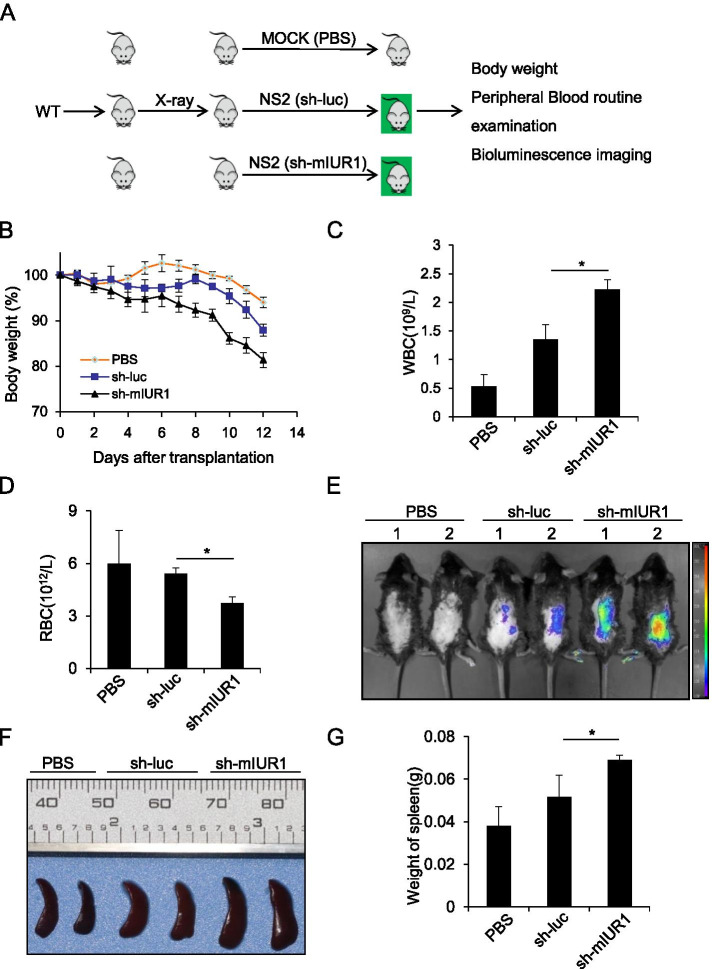
**Silencing murine lncRNA-IUR1 in Abl-transformed cells promotes cell survival and the development of leukemia in mice. A-D** Scheme of in vivo leukemia transplant (**A**). Sub-lethally irradiated wild type (WT) C57BL/6 N mice were infused with GFP-positive NS2 cells expressing control (sh-luc) or lncRNA-IUR1 shRNA (sh-mIUR1), or equal volume of PBS. The body weight of indicated mice was monitored for a period of 12 days (**B**). Quantity of white blood cells (WBCs) (**C**) and red blood cells (RBCs) (**D**) in peripheral blood of indicated mice, was detected by blood routine examination. **E** Bioluminescent imaging analysis of the distribution of GFP-positive control (sh-luc) or lncRNA-IUR1 knockdown (sh-mIUR1) NS2 cells in sub-lethally irradiated C57BL/6 N mice at the 12th day after in vivo leukemia transplantation. PBS group was the negative control. Shown were representative images from at least three independent experiments with similar results. **F**, **G** Shown were representative images of spleens from indicated mice at the 12th day after in vivo leukemia transplantation (**F**). Weight of spleens from indicated mice were measured at the 12th days after in vivo transplantation (**G**)

To further address the functional involvement of lncRNA-IUR1 in Abl-induced leukemia, we generated a leukemia mouse model by injecting sub-lethally irradiated mice with control or lncRNA-mIUR1 knockdown GFP-positive NS2 cells stably expressing control or lncRNA-mIUR1 shRNA respectively (Fig. 5A). The effect of lncRNA-mIUR1 deficiency on Abl-mediated leukemia was then examined. As shown in Fig. 5B, the body weight of mice injected with lncRNA-mIUR1 knockdown NS2 cells lost much faster than that of mice challenged with control cells. Accordingly, the number of white blood cell (WBC) in the peripheral blood from mice injected with lncRNA-mIUR1 knockdown cells, significantly increased compared with that from mice treated with control cells, and meanwhile the number of red blood cell (RBC) decreased (Fig. 5C-D). No significant difference of platelet (PLT) was observed between mice treated with control or lncRNA-mIUR1 knockdown cells (Supplementary Fig. S4).

In addition, we examined the intensity of GFP signal in the whole body, and found that the GFP signal was much stronger in mice injected with lncRNA-mIUR1 knockdown NS2 cells than that in mice treated with control cells (Fig. 5E). Accordingly, spleens of mice injected with lncRNA-mIUR1 knockdown cells displayed obvious splenomegaly compared with that of mice treated with control cells (Fig. 5F, G). These observations demonstrate that loss of lncRNA-IUR1 promotes Abl-transformed cell survival and the development of Abl-mediated leukemia in mice.

### Knockout of murine lncRNA-IUR1 in mice facilitates Abl-mediated transformation of primary bone marrow cells and leukemia formation in mice

**Fig. 6:**
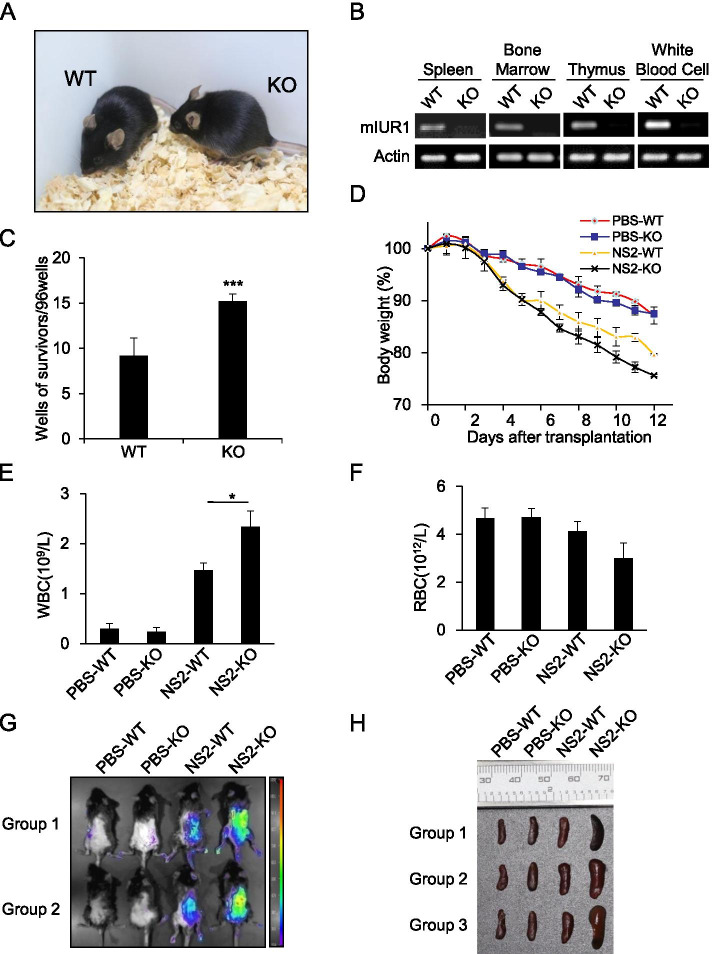
**Knockout of murine lncRNA-IUR1 in mice facilitates Bcr-Abl-mediated transformation of primary bone marrow cells, Abl-transformed cell survival and the development of leukemia in mice. A** The photo of lncRNA-IUR1 knockout (KO) mice and their WT littermate. **B** RT-PCR was performed to examine the expression of lncRNA-mIUR1 in the spleen, bone marrow cells (BMCs), thymus and white blood cells (WBCs) from WT or lncRNA-mIUR1 KO mice. **C** Primary bone marrow cells derived from WT or lncRNA-IUR1 KO mice, were infected with the retrovirus carrying Bcr-Abl oncogene, and the efficiency of transformation was measured by counting the number of Bcr-Abl-transformed cell clones. Data are presented as mean ± SEM. *n* = 3, ***p < 0.001. **D–F** Sub-lethally irradiated lncRNA-IUR1 KO and WT mice were injected with GFP-positive NS2 cells, or equal volume of PBS. The body weight of indicated mice was monitored for a period of 12 days (**D**). Quantity of WBCs (**E**) and RBCs (**F**) in peripheral blood of indicated mice, was detected by blood routine examination. **G** Bioluminescent imaging analysis of the distribution of GFP-positive NS2 cells in sub-lethally irradiated lncRNA-IUR1 KO and WT mice at the 12th day after in vivo leukemia transplantation. PBS group was the negative control. Shown were representative images from at least three independent experiments with similar results. **H** Shown were representative images of spleens from indicated mice at the 12th day after in vivo leukemia transplantation

To determine the role of lncRNA-IUR1 in malignant transformation by Abl oncogenes under a more physiological circumstance, we generated lncRNA-mIUR1 knockout (KO) mice (Fig. 6A, Supplementary Fig. S5A-B). The deficiency of lncRNA-mIUR1 was confirmed in multiple organs of the lncRNA-mIUR1 KO mice, including the spleen, bone marrow cells, thymus, and white blood cells from peripheral blood (Fig. 6B). Then, primary bone marrow cell (BMC) derived from wild type (WT) or lncRNA-mIUR1 KO mice, were infected with the retrovirus expressing Bcr-Abl oncogene, and the efficiency of transformation was measured by counting the number of Bcr-Abl-transformed cell clones. As shown in Fig. 6C, the clone number of Bcr-Abl-transformed BMCs from lncRNA-mIUR1 KO mice was significantly increased as compared with that of Bcr-Abl-transformed BMCs from WT mice, suggesting that knockout of murine lncRNA-IUR1 facilitated Bcr-Abl-mediated primary bone marrow transformation.

Next, we established the leukemia model using lncRNA-mIUR1 KO mice, and evaluated the effect of lncRNA-mIUR1 knockout on Abl-induced leukemia in mice. Sub-lethally irradiated WT or lncRNA-mIUR1 KO mice were injected with GFP-positive NS2 cells, and the development of Abl-mediated leukemia was examined (Supplementary Fig. S5C). As shown in Fig. 6D, after injection with NS2 cells, the body weight of lncRNA-mIUR1 KO mice lost much faster than that of WT mice. The number of WBC in the peripheral blood from lncRNA-mIUR1 KO mice was increased remarkably compared to that from WT mice, and meanwhile the number of RBCs decreased (Fig. 6E, F). No significant difference of PLT was observed between WT and lncRNA-mIUR1 KO mice challenged with NS2 cells (Supplementary Fig. S5D). Moreover, we examined the intensity of GFP signal in the whole body, and observed that the GFP signal in lncRNA-mIUR1 KO mice injected with NS2 cells, was much stronger than that in challenged WT mice (Fig. 6G). Additionally, spleens of lncRNA-mIUR1 KO mice displayed obvious splenomegaly compared with that of WT mice (Fig. 6H, Supplementary Fig. S5E). Collectively, these results reveal that knockout of lncRNA-IUR1 in mice facilitates Abl-mediated primary bone marrow transformation and leukemia formation in mice.

### LncRNA-IUR1 negatively regulates STAT5-mediated GATA3 expression

**Fig. 7:**
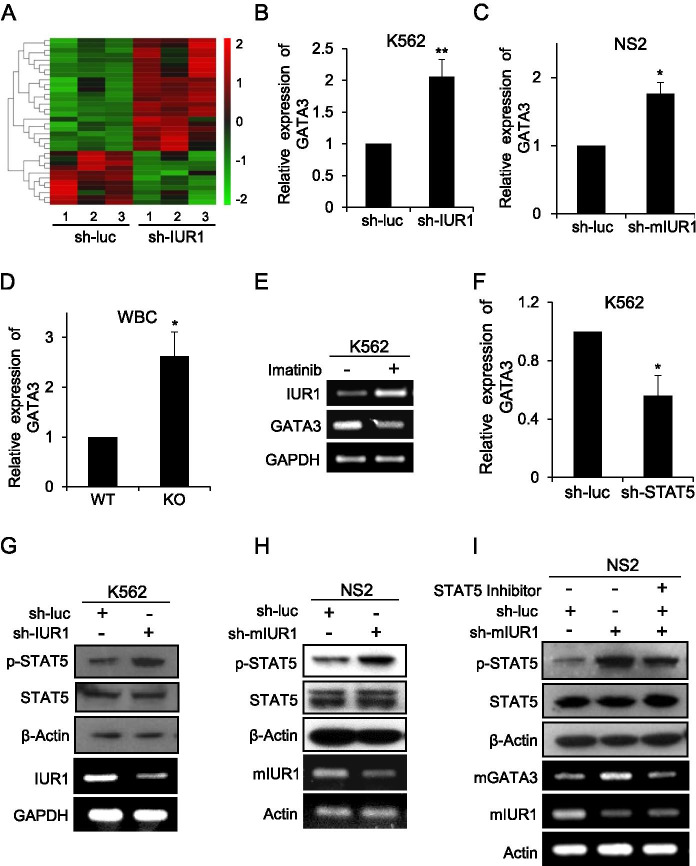
**LncRNA-IUR1 negatively regulates STAT5-mediated GATA3 expression in Abl-positive leukemic cells. A** Transcriptome RNA sequencing analysis of control (sh-luc) and lncRNA-IUR1 knockdown (sh-IUR1) K562 cells. **B, C** Quantitative real-time PCR analysis of GATA3 mRNA levels in control and lncRNA-IUR1 knockdown K562 (**B**) or NS2 (**C**) cells. Data are presented as mean ± SEM. n = 3, *p < 0.05, **p < 0.01. **D** Quantitative real-time PCR was performed to examine GATA3 mRNA levels in white blood cells (WBCs) derived from lncRNA-IUR1 KO and WT mice. Data are presented as mean ± SEM. n = 3, *p < 0.05. **E** RT-PCR was performed to examine the expression of GATA3 and lncRNA-IUR1 in K562 cells treated with imatinib. **F** Quantitative real-time PCR analysis of GATA3 mRNA levels in control and STAT5 knockdown K562 cells. Data are presented as mean ± SEM. n = 3, *p < 0.05. **G**, **H** Phosphorylation levels of STAT5 were examined in control and lncRNA-IUR1 knockdown K562 (**G**) or NS2 (**H**) cells by Western Blotting. **I** Western Blotting was performed to detect phosphorylation levels of STAT5 in control and lncRNA-IUR1 knockdown NS2 cells treated with STAT5 inhibitor

To decipher the molecular mechanism by which lncRNA-IUR1 regulates Abl-mediated cellular transformation, we performed RNA sequencing (RNA-Seq) to analyze the differential expression of genes in control and lncRNA-IUR1 knockdown K562 cells (Fig. 7A). Of particular interest, the expression of GATA3, a critical transcription factor involved in multiple cell processes including T-cell differentiation, tumor progression and metastasis [38, 39], significantly increased in lncRNA-IUR1 knockdown K562 cells compared with the controls (Fig. 7A). Notably, it has been shown that GATA3 could promote leukemic transformation by driving MYC enhancer activity, and inherited GATA3 variants are associated with Ph-like childhood acute lymphoblastic leukemia and risk of relapse [38, 39], suggesting a possibility that lncRNA-IUR1 is involved in Abl-induced tumorigenesis through regulating GATA3 expression.

To test this possibility, we first determined whether the expression of GATA3 was regulated by lncRNA-IUR1 in Abl-transformed leukemic cells. Indeed, the expression of GATA3 vastly increased in lncRNA-IUR1 knockdown K562 cells, whereas lncRNA-IUR1 overexpression led to a significant decrease of GATA3 levels in the cells (Fig. 7B and Supplementary Fig. S6A). Disruption of lncRNA-mIUR1 expression in v-Abl-transformed NS2 cells, also led to a significant increase of GATA3 expression (Fig. 7C). Besides, the level of GATA3 in WBCs and BMCs derived from lncRNA-IUR1 knockout mice was obviously higher than that in these cells from WT mice (Fig. 7D, Supplementary Fig. S6B). In addition, expression of GATA3 was markedly decreased in imatinib-treated K562 cells in which the expression of lncRNA-IUR1 was highly induced (Fig. 7E). These results indicate that lncRNA-IUR1 negatively regulates GATA3 expression in Abl-transformed leukemic cells.

STAT5 has been reported as a transcription factor of GATA3 [40], which prompted us to probe whether STAT5 contributes to the expression of GATA3 in Abl-positive leukemic cells. As shown in Fig. 7F, GATA3 RNA levels were significantly decreased in STAT5 knockdown K562 cells, suggesting that STAT5 is required for the transcription of GATA3 in Abl-transformed cells. Therefore, we next asked whether STAT5 is involved in lncRNA-IUR1-mediated regulation of GATA3 in Abl-positive leukemic cells. To this end, we examined the effect of altering lncRNA-IUR1 expression on STAT5 activation in Abl-transformed cells. Indeed, elevated phosphorylation levels of STAT5 were observed in lncRNA-IUR1 depleted Abl-transformed cells, whereas overexpression of lncRNA-IUR1 caused an obvious reduction of STAT5 phosphorylation in cells (Fig. 7G and H, Supplementary Fig. S6C-E). Importantly, the increased GATA3 expression caused by depletion of lncRNA-IUR1 in the cells, could be reversed by the treatment with STAT5 inhibitor (Fig. 7I). In addition, we also evaluated the effect of lncRNA-IUR1 on the JAK2/STAT3 signaling. Neither depletion nor forced expression of lncRNA-IUR1 had significant effect on the phosphorylation of JAK2 and STAT3 in K562 cells (Supplementary Fig. S6F-G). These experiments demonstrate that lncRNA-IUR1 negatively regulates GATA3 expression through suppression of STAT5 activation.

### LncRNA-IUR1 inhibits Abl-induced tumorigenesis by suppressing GATA3 expression

**Fig. 8:**
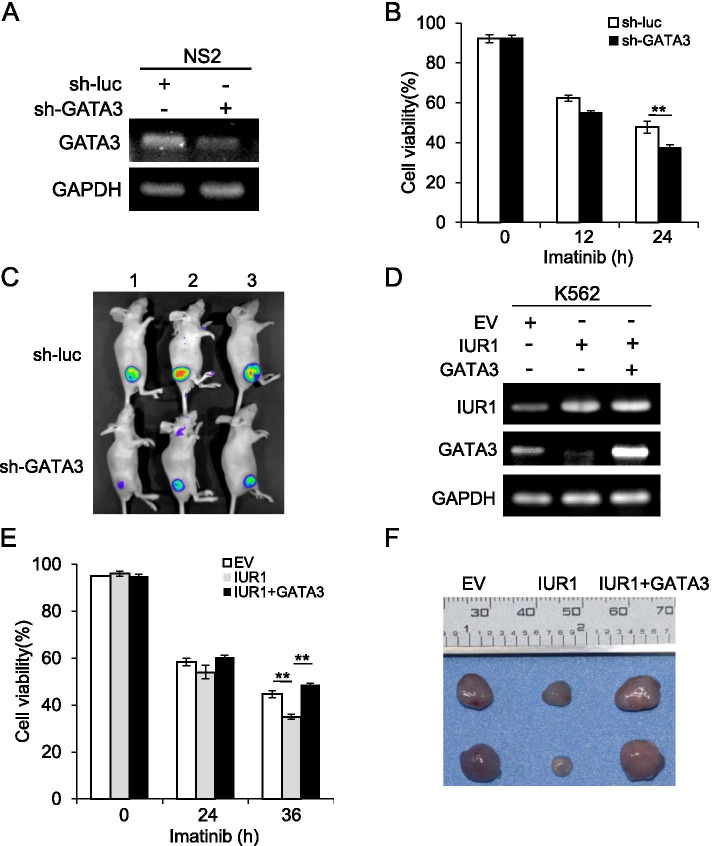
**LncRNA-IUR1 dampens Abl-induced tumorigenesis by suppressing GATA3 expression. A** RT-PCR was performed to examine GATA3 mRNA levels in NS2 cells stably expressing control (sh-luc) or GATA3 shRNA (sh-GATA3). **B** Cell viability of control and GATA3 knockdown NS2 cells, was analyzed by flow cytometry upon treatment with imatinib (2.5 μM). Data are presented as mean ± SEM. n = 3, **p < 0.01. **C** Nude mice were subcutaneously injected with control or GATA3 knockdown NS2 cells. Tumor growth was measured by bioluminescent imaging. Shown were representative images from at least three independent experiments. **D** RT-PCR was performed to examine lncRNA-IUR1 and GATA3 RNA levels in K562 cells stably expressing empty vector (EV), lncRNA-IUR1, or lncRNA-IUR1 and GATA3. **E** Cell survival analysis of K562 cells stably expressing EV, lncRNA-IUR1, or lncRNA-IUR1 and GATA3 in response to imatinib treatment (5 μM). Data are presented as mean ± SEM. n = 3, **p < 0.01. (**F**) Nude mice were subcutaneously injected with K562 cells stably expressing EV, lncRNA-IUR1, or lncRNA-IUR1 and GATA3. Shown were representative images of tumors excised from these nude mice

Next, we asked whether lncRNA-IUR1 suppresses Abl-induced tumorigenesis through regulating GATA3 expression. To test this, we first evaluated the role of GATA3 in Abl-mediated transformation. Control and GATA3 knockdown NS2 cells stably expressing control or GATA3 shRNA were generated, treated with imatinib, and subjected to cell survival analysis (Fig. 8A). We observed that proportion of viable cells in GATA3 knockdown cells was significantly reduced as compared with that in control cells after imatinib treatment, suggesting that GATA3 is associated with Abl-transformed cell survival in response to imatinib treatment (Fig. 8B). Then, we further investigated the effect of GATA3 depletion on Abl-induced tumorigenesis in vivo. Control or GATA3 knockdown NS2 cells were injected into nude mice subcutaneously, and tumor growth was examined. As expected, tumors formed by GATA3 knockdown cells grew much slower than that formed by control cells (Fig. 8C). Moreover, we observed that overexpression of GATA3 promoted cell survival of Abl transformants upon imatinib treatment (Supplementary Fig. S7A-B). These results demonstrate that GATA3 is required for efficient tumorigenesis induced by Abl oncogenes.

Then, we further investigated the involvement of GATA3 in regulation of Abl-induced tumorigenesis by lncRNA-IUR1. We generated K562 cells stably overexpressing empty vector (EV), lncRNA-IUR1, or combination of lncRNA-IUR1 and GATA3. These cells were treated with imatinib, and subjected to cell survival analysis (Fig. 8D, Supplementary Fig. S7C). As shown in Fig. 8E, less viable cells were detected in lncRNA-IUR1 overexpressing K562 cells compared with that in control cells. Intriguingly, forced expression of GATA3 in lncRNA-IUR1 overexpressing K562 cells can rescue the decreased cell survival caused by enhanced expression of lncRNA-IUR1. In line with these results, tumors formed by lncRNA-IUR1 overexpressing K562 cells grew much slower than that formed by control cells in the xenograft mouse model, whereas enhanced expression of GATA3 in lncRNA-IUR1 overexpressing cells can reverse the inhibitory effect of lncRNA-IUR1 overexpression on xenografted tumor growth in mice (Fig. 8F). Collectively, these results reveal that lncRNA-IUR1 inhibits Abl-induced tumorigenesis through suppression of GATA3 expression.

## Discussion

Cellular transformation mediated by *Abl* oncogenes is associated with tumorigenesis of several types of leukemia including CML**.** It is well known that Abl-induced tumorigenesis is a complicated process involving dysregulation of multiple signal pathways that regulate cell survival and proliferation. Although considerable progress has been made to address the molecular mechanisms accounting for Abl-mediated oncogenic transformation, the roles of lncRNAs in Abl-induced leukemia remain largely unexplored and attract increasing attention. In the present study, we identified an lncRNA IUR1 as a critical negative regulator of Abl-induced tumor formation. LncRNA-IUR1 is expressed at a very low level in Bcr-Abl-positive cells from chronic myeloid leukemia patients. Importantly, it was significantly induced in Abl-positive leukemic cells treated by imatinib. Depletion of lncRNA-IUR1 promoted Abl-positive leukemic cell survival and tumor growth in the xenograft mouse model, while enhanced expression of lncRNA-IUR1 sensitized cell to apoptosis and suppressed xenografted tumor growth in mice. Furthermore, we identified the mouse homologous lncRNA-IUR1. Knockout of lncRNA-mIUR1 in mice facilitated Abl-mediated transformation of primary bone marrow cells, Abl-transformed cell survival, and the development of leukemia in mice. These findings suggest that low expression of lncRNA-IUR1 is required for efficient tumorigenesis induced by Abl oncogenes. However, how Bcr-Abl downregulates the expression of lncRNA-IUR1 and evades from the inhibitory effect of lncRNA-IUR1 remain to be further defined.

Imatinib can competitively bind to the adenosine triphosphate (ATP) binding pocket of Bcr-Abl, thereby effectively inhibiting its tyrosine kinase activity [41]. Imatinib has been used for the frontline treatment for most chronic myeloid leukemia (CML) patients and has proven to be very effective [42]. The second-generation drugs targeting Bcr-Abl (dasatinib, nilotinib, and bosutinib) and the third-generation inhibitor ponatinib have been developed, since the emergence of resistance to imatinib has been one big problem for BCR-Abl–positive patients [43]. In this study, we found that expression of lncRNA-IUR1 was highly induced by imatinib treatment, indicating that induction of the negative regulator lncRNA-IUR1 may contribute to the effectiveness of imatinib in treating Bcr-Abl–positive patients. In turn, insufficient induction of lncRNA-IUR1 expression might be one mechanism underlying the resistance to imatinib. Actually, abnormal expression of lncRNAs has been linked to acquired resistance toward drug treatment. For instance, lncRNA TUG1 was upregulated in ADR (Adriamycin)-resistant AML (acute myelocytic leukemia) tissues and cells, and high expression of TUG1 was correlated with poor prognosis of AML patients [44]. LncRNA SNHG5 was up-regulated in imatinib resistant leukemic cells, and promoted imatinib resistance in CML. Overexpression of SNHG5 in imatinib-sensitive leukemic cells increased imatinib resistance and depletion of SNHG5 reduced cellular resistance to imatinib [45]. Therefore, it is of importance to elucidate the mechanism accounting for the upregulation of lncRNA-IUR1 induced by imatinib treatment, which may help to explore potential targeted therapies to treat imatinib-resistant Bcr-Abl–positive patients through enhancing the expression of lncRNA-IUR1.

The STAT5 signaling is constitutively activated by Bcr-Abl in CML, leading to uncontrolled cell survival and proliferation, which is indispensable for the initial and maintenance of Bcr-Abl–positive leukemia [46, 47]. Our previous study revealed that Bcr-Abl can cause tyrosine phosphorylation of suppressors of cytokine signaling 1 and 3 (SOCS-1 and SOCS-3), two potent suppressors of JAK2/STAT5 pathway, thereby relieving the inhibitory effects of SOCSs on STAT5 activation and facilitating Bcr-Abl-mediated transformation [36]. In an attempt to explore the mechanism by which lncRNA-IUR1 regulates Abl-induced tumorigenesis, we found that lncRNA-IUR1 suppresses STAT5 activation in Abl-positive leukemic cells. The phosphorylation of STAT5 was highly reduced in lncRNA-IUR1 overexpressing Abl-transformed cells, while loss of lncRNA-IUR1 enhanced STAT5 phosphorylation in cells. Furthermore, we performed RNA-Seq to identify genes that may be involved in regulation of Abl-induced tumorigenesis by lncRNA-IUR1. We found that the expression of GATA3 was negatively regulated by lncRNA-IUR1, as evidenced by profound inhibitory effect of lncRNA-IUR1 overexpression on GATA3 expression in Abl-positive leukemic cells. GATA3 has been reported as a critical transcription factor involved in multiple cell processes, including tumor progression and metastasis [48–51]. Notably, it has been shown that GATA3 could promote leukemic transformation by driving MYC enhancer activity, and inherited GATA3 variants are associated with Ph-like childhood acute lymphoblastic leukemia and risk of relapse (40, 41). Our results show that GATA3 is required for Abl-transformed leukemic cell survival in vitro and xenografted tumor growth in vivo, revealing a critical role for GATA3 in Abl-induced tumorigenesis through regulating leukemic cell survival. Moreover, we found that lncRNA-IUR1 suppressed Abl-induced tumorigenesis through downregulating the expression of GATA3. More interestingly, it has been reported that STAT5 is required for the induction of GATA3 expression by IL-33 and IL-2 [40]. This prompted us to evaluate the involvement of STAT5 in lncRNA-IUR1-mediated regulation of GATA3. Indeed, the increased expression of GATA3 caused by depletion of lncRNA-IUR1 was reversed upon treatment with STAT5 inhibitor. This indicates that STAT5 signaling was employed by lncRNA-IUR1 to accomplish its regulation of GATA3, thereby suppressing Abl-induced tumorigenesis. LncRNAs could function through the motifs embedded in their sequences that enable the specific association between lncRNAs and DNA, RNA or protein [52–54]. Therefore, it is likely that lncRNA-IUR1 may interact with STAT5 and thereby suppress STAT5 activation. In turn, it is also possible that the JAK2/STAT5 signaling contributes to the downregulation of lncRNA-IUR1 in Abl-positive leukemic cells. These deserve further investigations in the future.

In this study, lncRNA-IUR1 knockout mouse model was generated and employed to evaluate the functional relevance of lncRNA-IUR1 in Abl-induced leukemia under a more sophisticated and physiological circumstance. Loss of murine lncRNA-IUR1 promoted Abl-mediated transformation of primary bone marrow cells, leukemic cell survival, and the development of Abl-mediated leukemia in mice, supporting that lncRNA-IUR1 acted as a key negative regulator of Abl-induced tumorigenesis. Longitudinal analysis in a large cohort of CML patients is necessary to address the association between CML patient survival and the expression of lncRNA-IUR1, and the relationship of lncRNA-IUR1 with imatinib resistance in CML patients, and further to evaluate the translational potential of lncRNA-IUR1 for diagnosis and treatment of Bcr-Abl–positive leukemia. These deserve further studies in the future.

## Conclusions

In summary, this study identified lncRNA-IUR1 as a critical negative regulator of Bcr-Abl-induced tumorigenesis. Deficiency of lncRNA-IUR1 promoted Abl-transformed cell survival and development of leukemia in mice. Mechanistically, lncRNA-IUR1 suppressed Bcr-Abl-induced tumorigenesis through negatively regulating STAT5-mediated GATA3 expression. These findings reveal the vital involvement and physiological significance of lncRNAs in Abl-mediated oncogenic transformation, and provide new insights into molecular mechanisms underlying Abl-induced leukemogenesis.

## Supplementary Information

**ESM 1.**

## Data Availability

All data generated or analyzed during this study are included in this published article, and its supplementary information files. The lncRNA cDNA microarray and RNA sequencing data from this study have been submitted to the NCBI Database of GEO Datasets under accession number GSE119770 and GSE181535 respectively. The sequence of murine lncRNA-IUR1 has been submitted to GenBank (MZ643464).

## Abbreviations

CML: chronic myeloid leukemia
ALL: acute lymphoblastic leukemia
TKIs: tyrosine kinase inhibitors
JAK/STAT: Janus family tyrosine kinase/signal transducer and activator of transcription
SOCS: suppressor of cytokine signaling
PI3K/AKT: phosphatidylinositol 3-kinase/protein kinase B
PTEN: phosphatase and tensin homolog
LncRNAs: long noncoding RNAs
IUR1: imatinib-upregulated lncRNA 1
ORF: open reading frame
CPC: coding potential calculator
ceRNA: competitive endogenous RNA
BMCs: bone marrow cells
PBCs: peripheral blood cells
PLT: platelet
RBCs: red blood cells
WBCs: white blood cells
shRNA: short hairpin RNA
