# Supplementary material for: A novel imatinib-upregulated long noncoding RNA plays a critical role in inhibition of tumor growth induced by Abl oncogenes
Source: Mol Cancer. 2022 Jan 3;21:5. doi: 10.1186/s12943-021-01478-5 (PMC8722111; doi:10.1186/s12943-021-01478-5)

SUPPLEMENTARY FIGURE LEGENDS

Figure S1. LncRNA-IUR1 is identified as a novel lncRNA that is induced by imatinib treatment

(A) A schematic representation of the genomic location of lncRNA-IUR1 and its adjacent genes *P2ry2* and *Fchsd2*. The orientation of arrows indicated the transcription direction.

(B) Agarose gel analysis of 3' RACE PCR products of lncRNA-IUR1 and 3' end sequence was show in below.

(**C**) LncRNA-IUR1 was cloned into pNL vector with an N-terminal Flag tag in three reading frames. The constructs were transfected into HEK293T cells for 48 h. Cell lysates were harvested and subjected to Western blotting with Flag antibody. Flag-gag was served as a positive control.

Figure S2. Altering lncRNA-IUR1 expression regulates Bcr-Abl-transformed cell-induced tumor growth in mice

(A) Cell cytotoxicity analysis of control and lncRNA-IUR1 knockdown K562 cells upon imatinib treatment. Data are presented as mean ± SEM. n = 3, ***p < 0.001.

(B) Cell survival of control and lncRNA-IUR1 knockdown K562 cells in response to imatinib treatment was analyzed by colony formation assay. Data are presented as mean ± SEM. n = 3, **p < 0.01.

(C) The Sub-G1 cells in control and lncRNA-IUR1 knockdown K562 cells after imatinib treatment, were examined by flow cytometry analysis. Data are presented as mean ± SEM. n = 3, *p < 0.05.

(D) TUNEL analysis of control and lncRNA-IUR1 knockdown K562 cells in response to imatinib treatment. Data are presented as mean ± SEM. n = 3, ***p < 0.001.

(E) Cell cycle progression of control and lncRNA-IUR1 knockdown K562 cells was analyzed by flow cytometry.

(F) Western blotting analysis of p53 protein levels in HEK293T cells, and control or lncRNA-IUR1 knockdown K562 cells with or without imatinib treatment.

(**G**) The levels of cleaved caspase-3, caspase-8, caspase-9 and PARP in control or

lncRNA-IUR1 knockdown K562 cells treated with or without imatinib, were detected by Western blotting.

(H-J) Immunohistochemistry of TUNEL **(H)**, cleaved caspase-3 **(I)** and Ki-67 **(J)** signals from tumors formed by control or lncRNA-IUR1 knockdown K562 cells in nude mice. Nuclei were visualized with DAPI (blue).

(K) LncRNA-IUR1 expression in tumors formed by control and lncRNA-IUR1 knockdown K562 cells was examined by RT-PCR.

(L) Cell cycle progression of control and lncRNA-IUR1 overexpressing K562 cells was analyzed by flow cytometry.

(M) Nude mice were subcutaneously injected with control or lncRNA-IUR1 overexpressing K562 cells. Tumor growth was monitored, and tumors were excised from the nude mice. Shown were representative images from at least three independent experiments.

Figure S3. Identification of murine homologous lncRNA-IUR1

(A) Sequence alignment analysis between human lncRNA-IUR1 transcript and the mouse genome, revealed a 442 bp mouse genome sequence with up to 72% homology to human lncRNA-IUR1.

(B) The CPC scores for murine lncRNA-IUR1 transcript in all three reading frames (http://cpc2.cbi.pku.edu.cn/).

(C) The protein-coding potential analysis of murine lncRNA-IUR1 was performed using ORF finder from NCBI.

Figure S4. Silencing murine lncRNA-IUR1 in Abl-transformed cells promotes cell survival and the development of leukemia in mice

Quantity of PLT in peripheral blood of mice infused with GFP-positive NS2 cells expressing control shRNA (sh-luc) or murine lncRNA-IUR1 shRNA (sh-mIUR1), or equal volume of PBS, was detected by blood routine examination.

Figure S5. Knockout of murine lncRNA-IUR1 in mice accelerates

Abl-transformed cell survival and the development of leukemia in mice

(A-B) Murine lncRNA-IUR1 knockout mice were generated by CRISPR/Cas9-based genome editing system. Two sgRNAs targeting 5' end (gRNA 1) and 3' end (gRNA 2) of murine lncRNA-IUR1 were respectively constructed and transcribed in vitro. The Cas9 mRNA and sgRNAs were co-injected into zygotes. The zygotes were then transferred into the oviduct of pseudo-pregnant ICR females at 0.5 dpc to farrow litters **(A)**. The litters were genotyped by PCR using primers including KO-mIUR1-F, KO-mIUR1-R and WT-F **(B)**.

(C) Scheme of *in vivo* leukemia transplant using lncRNA-IUR1 KO and WT mice. Briefly, lncRNA-IUR1 KO mice and their WT littermates were infused with GFP-positive NS2 cells or PBS by vena caudalis after sub-lethal irradiation. Experimental groups were labeled as NS2-KO and NS2-WT, and control groups were labeled as PBS-KO and PBS-WT.

(D) Quantity of PLT in peripheral blood of lncRNA-IUR1 or WT mice infused with GFP-positive NS2 cells, or equal volume of PBS, was detected by blood routine examination.

(E) Weight of spleens from indicated mice were measured at the 12th days after *in vivo* transplantation.

Figure S6. LncRNA-IUR1 negatively regulates STAT5-mediated GATA3 expression in Abl-positive leukemic cells

(A) Quantitative real-time PCR analysis of GATA3 mRNA levels in control and lncRNA-IUR1 overexpressing K562 cells. Data are presented as mean ± SEM. n = 3, *p < 0.05.

(B) Quantitative real-time PCR was performed to examine GATA3 mRNA levels in bone marrow cells (BMCs) derived from lncRNA-IUR1 KO and WT mice. Data are presented as mean ± SEM. n = 3, *p < 0.05.

(C-E) Phosphorylation levels of STAT5 were examined in control and lncRNA-IUR1 overexpressing K562 **(C)**, NS2 **(D)** or W44 **(E)** cells by Western Blotting.

(F-G) Western Blotting analysis of phosphorylation levels of JAK2 and STAT3 in

lncRNA-IUR1 overexpressing **(F)** or knockdown **(G)** K562 cells.

Figure S7. LncRNA-IUR1 inhibits Abl-induced tumorigenesis by suppressing GATA3 expression

(A) RT-PCR was performed to examine GATA3 mRNA levels in K562 cells expressing empty vector (EV) or GATA3.

(B) Cell viability analysis of control and GATA3 overexpressing K562 cells in response to imatinib treatment. Data are presented as mean ± SEM. n = 3, *$p < 0.05$.

(C) Scheme of constructs expressing EV, lncRNA-IUR1, or lncRNA-IUR1 and GATA3.

Supplementary Figure S1

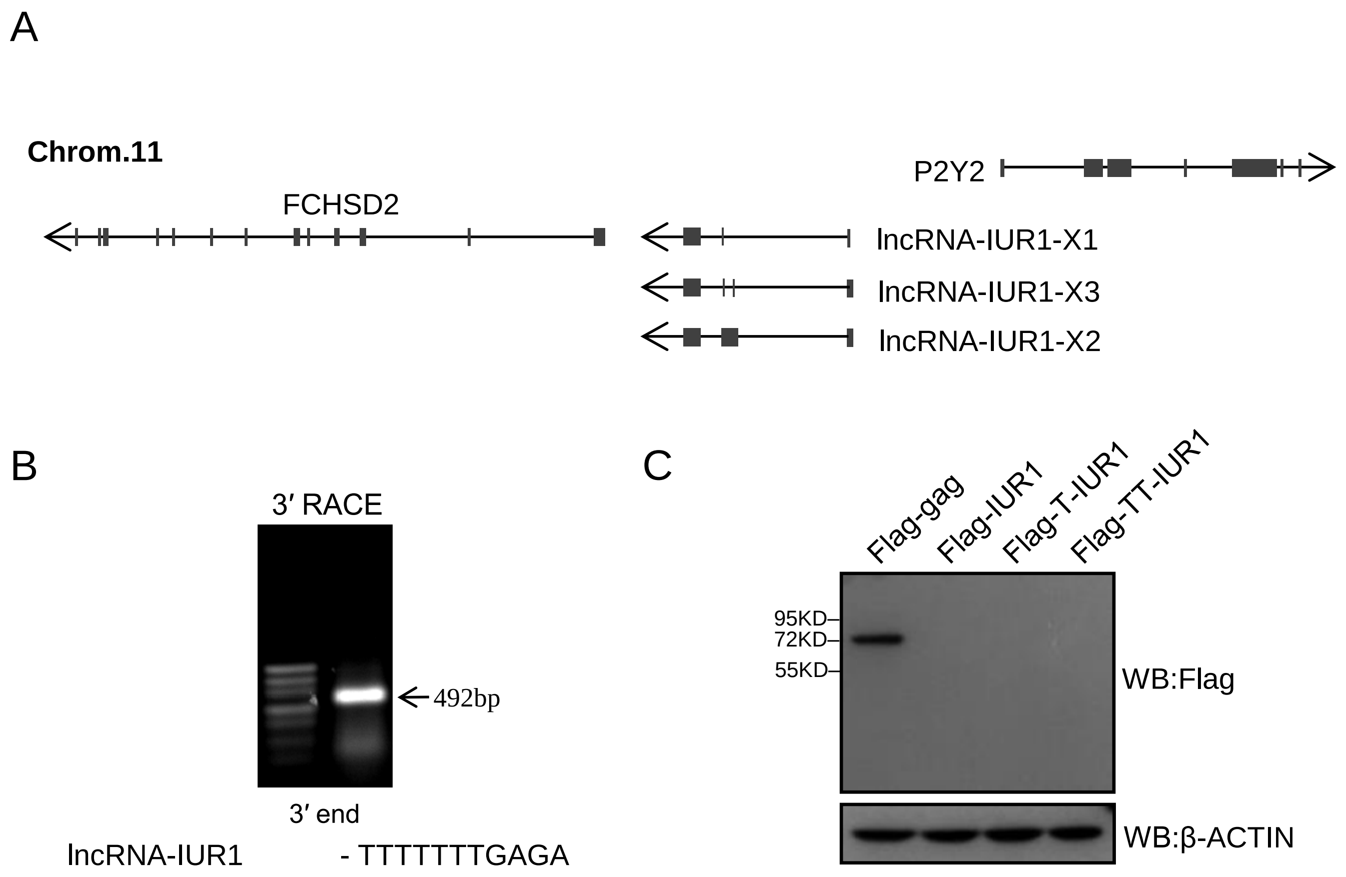

Supplementary Figure S2

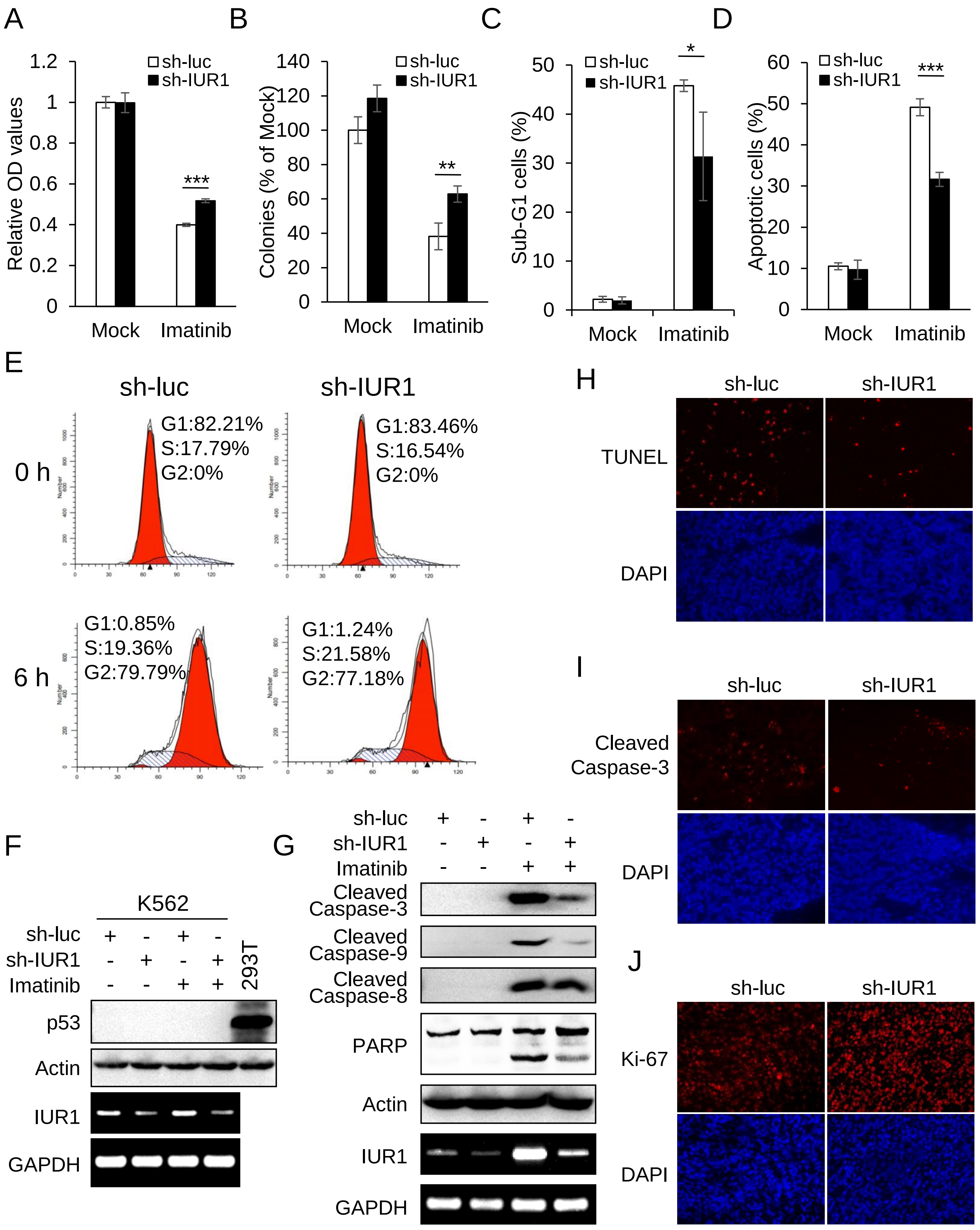

Supplementary Figure S2

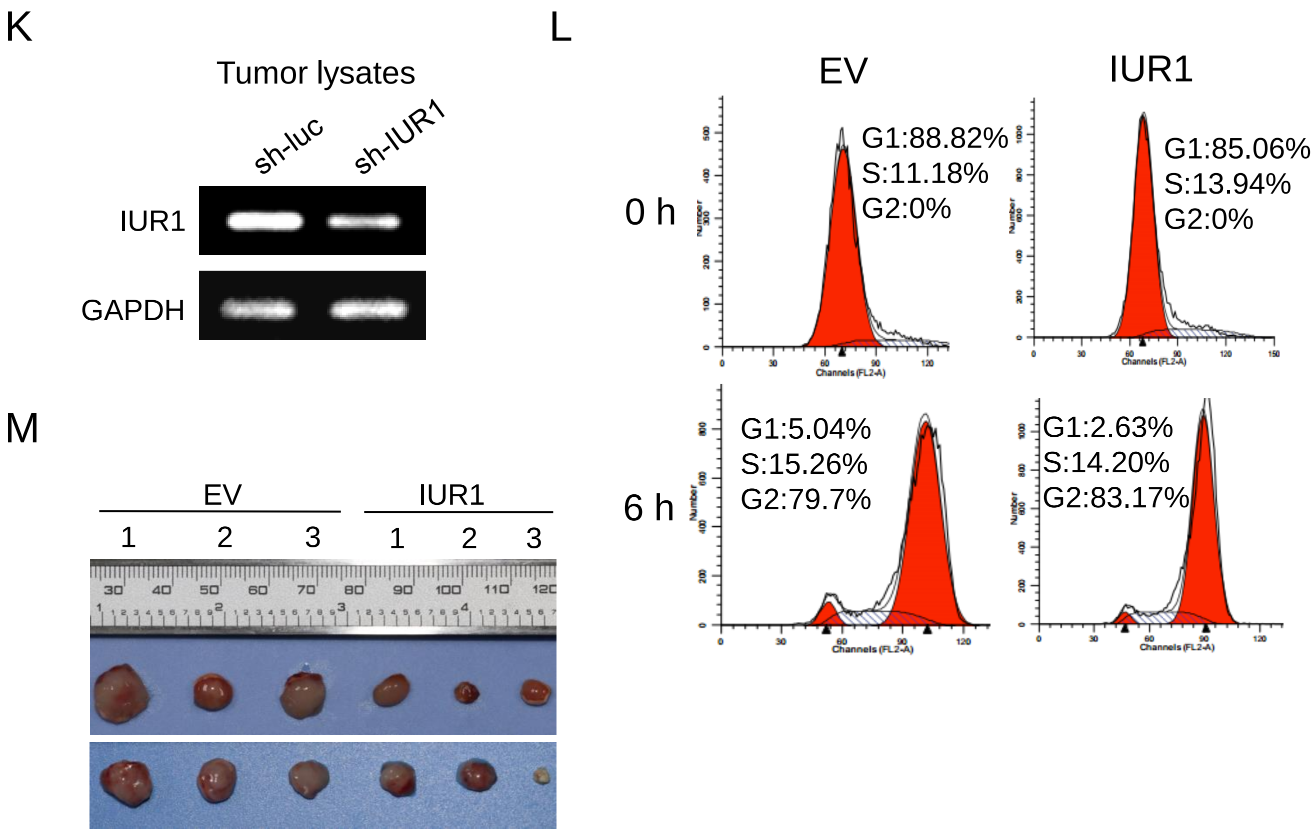

Supplementary Figure S3

A

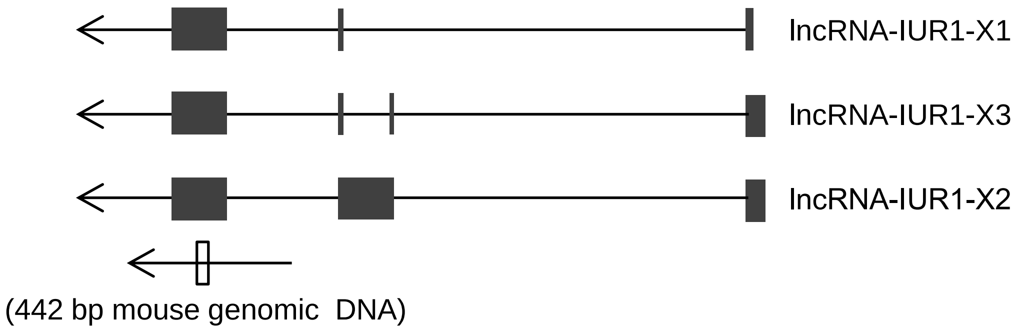

B

**Seq ID**	**C/NC**	**CODING POTENTIAL**
lncRNA-mIUR1	noncoding	0.0203522
T+lncRNA-mIUR1	noncoding	0.0203522
TT+lncRNA-mIUR1	noncoding	0.0203522

C

lncRNA-mIUR1

Label	Strand	Frame	Start	Stop	Length (nt | aa)
**ORF4**	**+**	**2**	**926**	**1273**	**348 | 115**
ORF1	+	1	421	702	282 | 93
ORF12	-	1	1566	1381	186 | 61
ORF13	-	1	1161	985	177 | 58
ORF11	-	1	1986	1819	168 | 55
ORF14	-	1	519	367	153 | 50
ORF5	+	2	1565	1711	147 | 48
ORF15	-	1	144	>1	144 | 47
ORF17	-	3	676	551	126 | 41
ORF8	+	3	2025	2132	108 | 35

Supplementary Figure S4

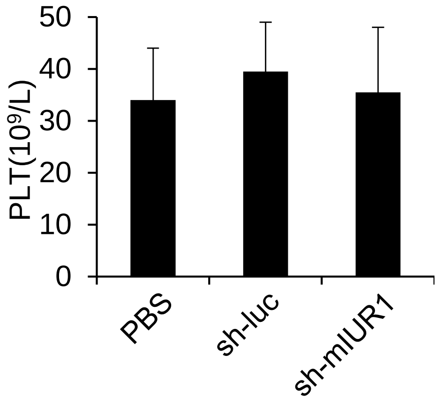

Supplementary Figure S5

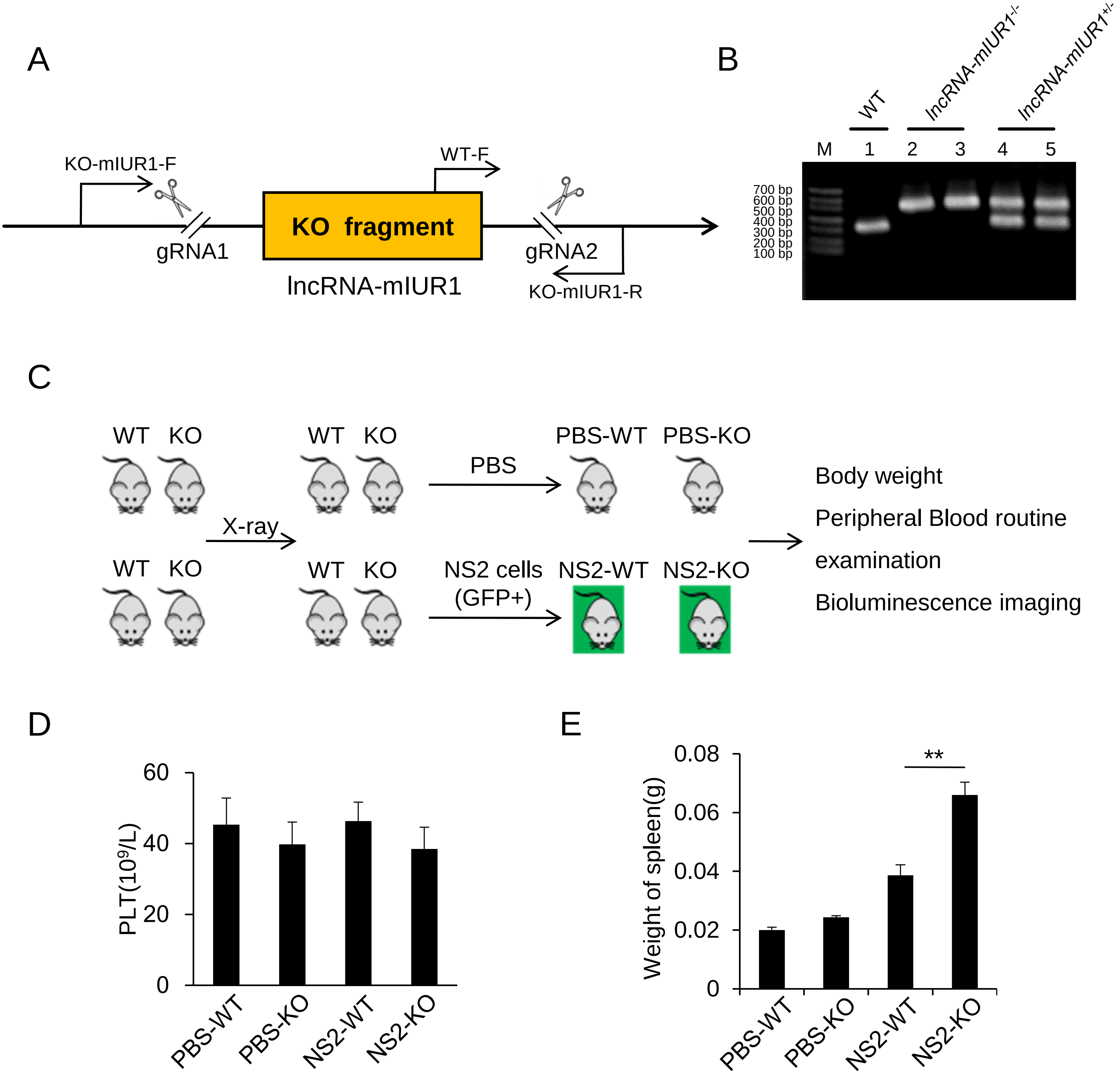

Supplementary Figure S6

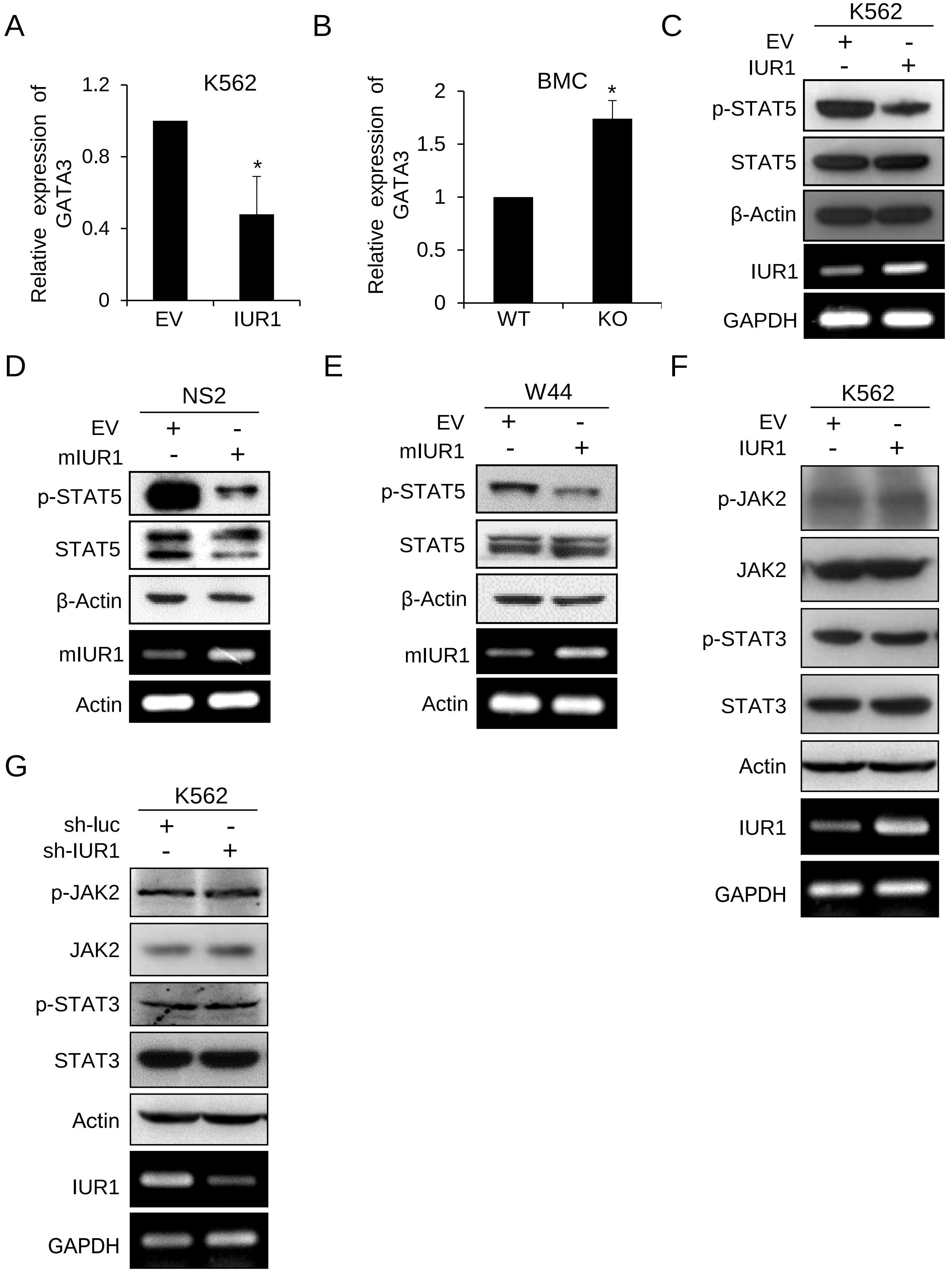

Supplementary Figure S7

A

B

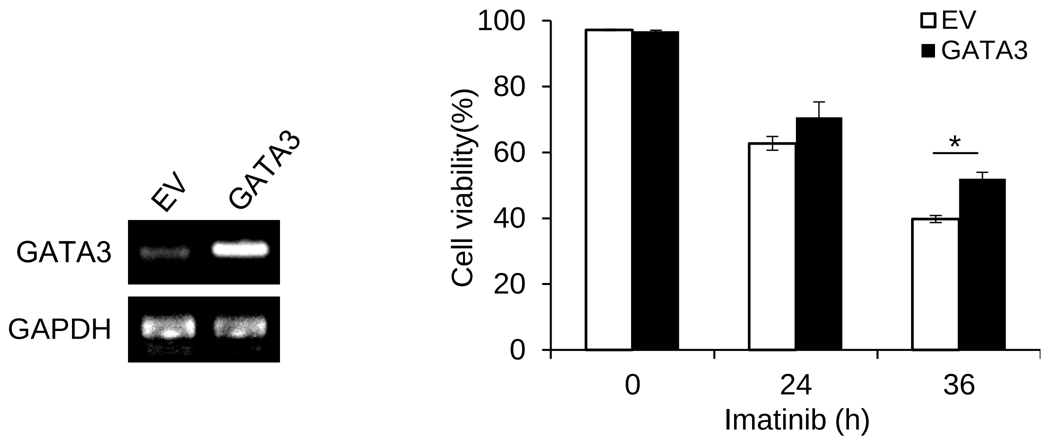

C

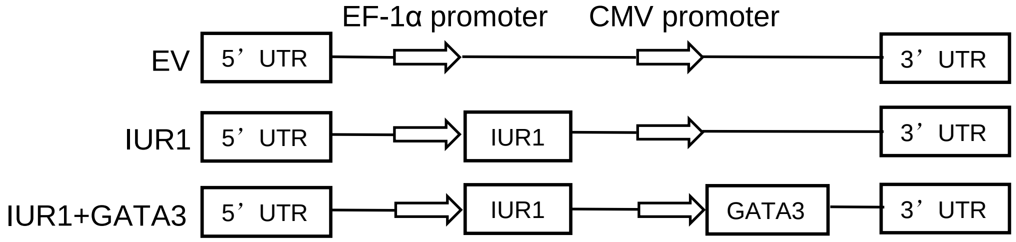

Supplement: Supplementary file 1 — ESM 1. [file 12943_2021_1478_MOESM1_ESM.pdf]
